# Atomically Precise Distorted Nanographenes: The Effect of Different Edge Functionalization on the Photophysical Properties down to the Femtosecond Scale

**DOI:** 10.3390/ma16020835

**Published:** 2023-01-15

**Authors:** Marco Reale, Alice Sciortino, Marco Cannas, Ermelinda Maçoas, Arthur H. G. David, Carlos M. Cruz, Araceli G. Campaña, Fabrizio Messina

**Affiliations:** 1 Dipartimento di Fisica e Chimica—Emilio Segrè, Università degli Studi di Palermo, Via Archirafi 36, 90123 Palermo, Italy; 2 Advanced Technologies Network Center, Università degli Studi di Palermo, Viale delle Scienze Ed. 18/A, 90128 Palermo, Italy; 3 Centro de Química Estrutural e Institute of Molecular Sciences, Instituto Superior Técnico, Universidade de Lisboa (Portugal), Av. Rovisco Pais 1, 1049-001 Lisboa, Portugal; 4 Department of Organic Chemistry, Unidad de Excelencia de Química (UEQ), Faculty of Sciences, University of Granada, Avda. Fuente Nueva s/n, 18071 Granada, Spain

**Keywords:** atomically precise nanographenes, fluorescent nanomaterials, distorted hexa-*peri*-hexabenzocoronene derivatives, optical properties

## Abstract

Nanographenes (NGs) have been attracting widespread interest since they combine peculiar properties of graphene with molecular features, such as bright visible photoluminescence. However, our understanding of the fundamental properties of NGs is still hampered by the high degree of heterogeneity usually characterizing most of these materials. In this context, NGs obtained by atomically precise synthesis routes represent optimal benchmarks to unambiguously relate their properties to well-defined structures. Here we investigate in deep detail the optical response of three curved hexa-*peri*-hexabenzocoronene (HBC) derivatives obtained by atomically precise synthesis routes. They are constituted by the same graphenic core, characterized by the presence of a heptagon ring determining a saddle distortion of their sp^2^ network, and differ from each other for slightly different edge functionalization. The quite similar structure allows for performing a direct comparison of their spectroscopic features, from steady-state down to the femtosecond scale, and precisely disentangling the role played by the different edge chemistry.

## 1. Introduction

Organic semiconductors (OSs) based on π-conjugated systems are considered very promising for their remarkable and stable optical properties and could play a key role in the development of next-generation optoelectronic devices [1,2]. In fact, OSs display several advantages with respect to their inorganic analogs since their carbon-based structure combines marked light absorption and photoluminescence features with mechanical flexibility, chemical versatility and high solution processability [3,4]. Among organic nanostructures, nanographenes (NGs) are attracting more and more interest because of their non-zero electronic bandgap and their strong visible photoluminescence, which can be tuned across the visible range, properties that greatly widen the range of their applications in optoelectronics [5,6] as compared to bulk graphene. In general, the term “nanographenes” stands for graphene fragments ranging from 1 to 100 nm in size [5]. Due to the confinement of their sp^2^ carbon network to the nanometric scale, their properties fall somewhere between those of graphene and polycyclic aromatic hydrocarbons (PAHs) molecules, with opto-electronic and spintronic properties that can be, in principle, modulated by modifying their structures, in terms of the size, shape, edges or chemical functionalization [7,8,9,10]. A paradigmatic example of NG is provided by hexa-*peri*-hexabenzocoronene (HBC), which is often regarded as the smallest NG [11]. 

In order to fully benefit from the advantages of NGs, it is of utmost importance to reach tight control over their structure, allowing a fine-tuning of their opto-electronic characteristics for applications. Top-down synthesis approaches allow easily obtaining large quantities of NGs, but do not guarantee adequate control over their structure, leading to quite heterogeneous materials [9,10,12,13,14] whose properties could be very different if studied at the ensemble or single-particle level [15]. In order to overcome this issue, bottom-up strategies based on rigorous organic synthesis routes permit obtaining well-defined NGs of precise structures designed with ultimate atomic accuracy [16]. Such tailored NGs are perfect benchmarks that can provide insight into the role of every specific structural modification on their fundamental opto-electronic properties. The possibility of finely tuning physico-chemical properties makes NGs extremely interesting also in view of applications in materials science. Indeed, a multitude of works on the design and characterization of NGs with controllable size [17,18], edge structure [19,20], nonplanarity [21,22] and functional groups [23,24] have been reported in recent literature. In particular, distorted NGs are lately attracting a special interest since their curved non-planar structure determines an increase in the solubility and processability in organic solvents [25,26] and prevents their aggregation through π-π interactions. To date, very sparse literature has been fostered to study the role of edge-functionalization on the photophysical properties of atomically precise nanographenes [24,27,28,29,30,31], and reports concerning π-conjugated curved systems are even more rare [32,33,34,35].

**Figure 1:**
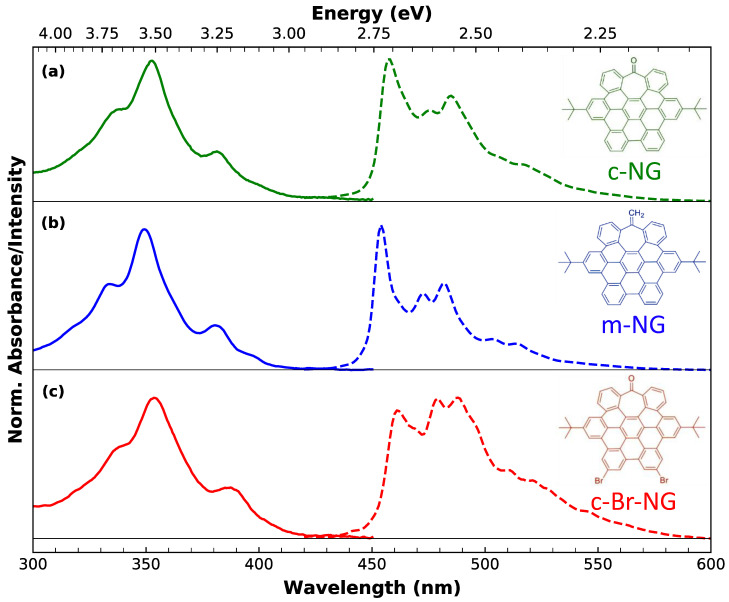
Normalized absorption (continuous lines) and photoluminescence (dashed lines) of (**a**) **c-NG**, (**b**) **m-NG** and (**c**) **c-Br-NG** in dichloromethane. The photoluminescence spectra were collected upon excitation at 350 nm. The corresponding chemical structure of each nanographene is reported on the right of the respective subpanel.

Here, through a side-by-side comparative study, we fully detailed the main optical properties of three atomically precise distorted NGs. The structures of our model systems can be pictured (Figure 1) as distorted HBC derivatives containing a seven-membered ring at the edge of their carbon network and different edge chemical groups. The deliberated inclusion of the heptagon ring in the HBCs [36] forces out from planarity their otherwise purely hexagonal networks, while the choice of different edge functional groups has a deep influence on the optical response of the NGs. These three model NGs were probed by a battery of steady-state and time-resolved spectroscopic techniques to fully reconstruct their optical photocycle down to the femtosecond scale and provide a comprehensive picture of the earliest excited-state relaxation dynamics. Supported by the precise knowledge of their structure, our photophysical characterization permits elucidating the impact that the different functional groups have on excited state dynamics of NGs, by means of precise structure–properties relationships offering promising routes to tailor NGs for specific future applications.

## 2. Materials and Methods

### 2.1. Synthetic Procedure

The heptagon-containing HBCs were synthesized according to previously reported strategies, following a cobalt-catalyzed alkyne cyclotrimerization reaction between functionalized benzophenones and diphenylacetylenes, and a subsequent Scholl reaction [36]. For **c-NG,** *tert*-butyl functionalized 2,2′-*bis*(phenylacetylene)-benzophenone and diphenylacetylene were used, while the exocyclic double bond of **m-NG** can be introduced by reaction of **c-NG** with Tebbe’s reagent [37]. **c-Br-NG** was prepared using 1,2-*bis*(4-bromophenyl)acetylene during the cobalt-catalyzed reaction [38]. 

### 2.2. Steady-State Spectroscopies

Absorption and photoluminescence spectra of the compounds were acquired in a liquid phase (dichloromethane as solvent) in a 1 cm cuvette. UV-Vis measurements in the 300–750 nm range were performed by means of a single-beam, optical fiber spectrophotometer (Avantes, Apeldoorn, The Netherlands) coupled to a dual halogen-xenon light lamp used as a source. For steady-state emission data collection, we used a FP6500 spectrofluorometer (JASCO Corporation, Tokyo, Japan) with a 3 nm bandwidth, a 150 W Xenon Lamp as an excitation source and a photomultiplier as a detector. Emission quantum yields of dichloromethane solutions of the compounds were calculated by comparing their emission intensity to that of a coumarin dye (QY = 94%) aqueous solution prepared at the same absorbing conditions. 

### 2.3. Time-Resolved Fluorescence

Time-resolved fluorescence data were acquired by means of a tunable laser system (Vibrant OPOTEK, Carlsbad, CA, USA) constituted by a Q-switched Nd:YAG laser whose third harmonic is used to pump an optical parametric oscillator. This excitation setup provides pulses of 5 ns duration with a repetition rate of 10 Hz. The energy/pulse was set to 40 μJ. The emission of the sample under study is dispersed into its spectral component by a prism and collected by an intensified CCD camera (PI-MAX Princeton Instruments, Trenton, NJ, USA) synchronized to the excitation laser. Data acquisition consists of recording the emission spectra by integrating the signal over a certain temporal window at different programmable delays from photoexcitation in order to reconstruct the kinetics of the entire decaying emission band. The obtained decay kinetics were least-squares fitted to exponential functions convoluted with a gaussian instrumental function accounting for the laser pulse temporal profile.

### 2.4. Femtosecond Transient Absorption

The femtosecond transient absorption measurements were acquired by using a home-built pump–probe setup. The beam of a 5 kHz Ti:Sapphire femtosecond amplifier (Spectra Physics Solstice-Ace, Milpitas, CA, USA), generating 45 fs pulses peaking at 800 nm, is split through a beam splitter (80%/20%) to generate the pump and probe pulses. The pump is obtained by frequency-doubling (type I phase-matching) the first branch of an 800 nm beam via a 250 μm beta-BBO crystal and isolated from the fundamental by a Schott BG40 filter. In order to produce the probe, the second branch of the 800 nm beam is focused on a 1 mm quartz cuvette containing D_2_O (spectral range 400–680 nm) or on a CaF_2_ crystal maintained under movement (spectral range 330–650 nm). A motorized delay stage allows for controlling the delay between the pump and the probe pulses, and a chopper is used to chop the pump beam at 2.5 kHz with the aim to alternatively record 5000 pumped and 5000 unpumped spectra for each delay. The pump and probe are focused and spatially overlapped by a parabolic mirror (f = 150 mm) on the sample, which is continuously made flown in a 200 μm thick flow cell by a peristaltic pump. The probe transmitted after the sample is dispersed by a Brewster-angle silica prism and focused by a lens on a single shot detector (Glaz-LineScan I, Synertronic Designs, Stellenbosch, South Africa), appropriately synchronized. The chosen configuration allows for the acquisition of transient absorption spectra with a spectral and temporal resolution of 3 nm and 90 fs, respectively. The data were acquired in a linear regime, with pump energy smaller than 50 nJ/pulse, and scanning over the pump–probe delay with 15–25 acquisitions. In order to work under the so-called magic angle conditions, the polarization of the pump beam was regulated by a waveplate. The reported measurements were corrected by standard procedures to eliminate the effects of cross-phase modulation and group velocity dispersion.

## 3. Results and Discussion

As can be appreciated from the chemical structures shown below in Figure 1, all three nanographenes share the same identical carbon core featured with two peripherical *tert*-butyl groups. If we consider the carbonyl functionalized nanographene **c-NG** (a) as a benchmark, the other two compounds differ for specific edge chemical modifications. More specifically, the oxygen of the peripherical tropone moiety of **c-NG** is replaced by a methylene group in **m-NG** (b). Instead, in **c-Br-NG** (c), two edge hydrogens are replaced by two bromine atoms. 

Figure 1 permits appreciating the well-defined fine structure characterizing both the steady-state absorption (continuous lines) and the emission (dashed lines) spectra of dichloromethane solutions of the three nanographenes (in 10^−6^ M concentration). UV-Vis spectra reveal essentially the same structured profile, with three main subpeaks at λ~355, 338 and 380 nm, which, according to Clar’s rule for PAHs [39], can be associated to $\beta^{\prime }$, β and p-bands. The very weak absorption edge between 430 and 460 nm, better discernable in more concentrated solutions (10^−5^ M) (see Figure S1), is assigned to α-bands. Its low oscillator strength accounts for the forbidden nature of such a lowest energy transition. Since the main absorption profile is substantially the same in all three nanographenes, we can infer that the different edge functionalization has a negligible impact on the main absorption transitions. 

The same solutions of nanographenes excited at 350 nm reveal blue photoluminescence (PL) exhibiting vibrationally resolved subpeaks. Differently from the absorption, the emission bandshape is highly sample-dependent, suggesting a deep influence of the functional groups on the lowest-energy emissive state only rather than on the upper electronic states. For each nanographene, the emission is totally independent of the excitation wavelength, as shown by the excitation-emission map (Figure S2). The highly fine-structured shape of absorption and emission bands, as well as the independent excitation behavior, demonstrates at the same time the high homogeneity of the samples and that the emission, for each of them, can be ascribed to a single chromophore and takes place from the lowest excited state, in compliance to Kasha’s rule. Moreover, the perfect overlap between the absorption and the excitation spectra further confirms the absence of impurities (Figure S3). 

If we look at **c-NG** as a representative case, a qualitative analysis of its emission bandshape allows us to recognize a zero-phonon line at 457 nm and a dominant vibronic progression (457, 485 and 517 nm), with a spacing of about 1300 cm^−1^ compatible with the C-C stretching mode. However, from the presence of other maxima, we can infer that at least another vibrational mode is associated with the emissive transition. A vibronic progression with ~1300 cm^−1^ spacing is also recognizable in the emission of **m-NG** and **c-Br-NG**, where the zero-phonon line position is located at 455 and 461 nm, respectively. Moreover, having proved that the emissive transition occurs from a single electronic state, the shape modifications of the finely structured emission attest that the vibronic coupling pathways of the emissive state, which determine the relative ratio between vibronic peaks belonging to the observed progressions, are quite affected by the different edge functionalization, particularly in the presence of the two bromine atoms. 

Furthermore, the variation in the edge chemistry has an even more marked impact on the emission quantum yields (QYs): 2%, 5% and 0.1% for **c-NG**, **m-NG** and **c-Br-NG,** respectively. By considering **c-NG** as a reference, the substitution of oxygen with a CH_2_ group determines a more than a two-fold increase of QY in **m-NG**, while the presence of two bromine atoms in **c-Br-NG** dramatically reduces the QY of an order of magnitude. Thus, the tunability of both the shape and quantum yield of the emission is an explicit indication that the controlled edge functionalization affects the emissive electronic state as well as the relaxation pathways.

**Figure 2:**
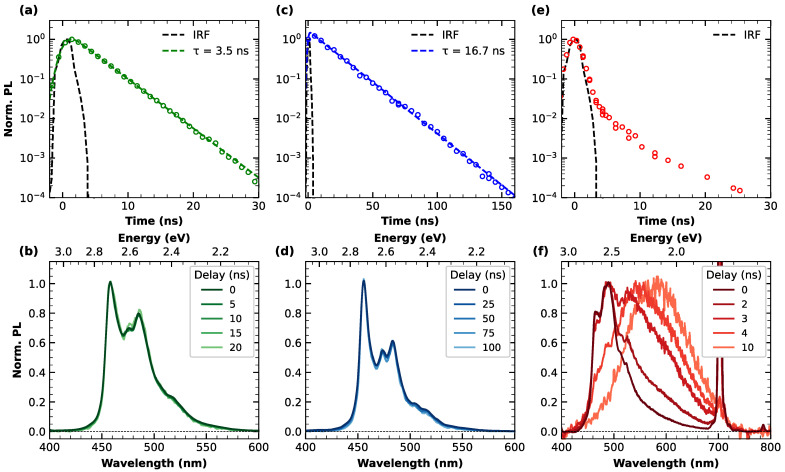
Top panels: normalized emission decay and respective least-squares fitting curves (dashed lines) of (**a**) **c-NG**, (**c**) **m-NG** and (**e**) **c-Br-NG**, acquired at $\lambda_{exc}=350$ nm and $\lambda_{em}=460\;$nm. Bottom panels: normalized emission spectra of (**b**) **c-NG**, (**d**) **m-NG** and (**f**) **c-Br-NG**, acquired at different delays from photoexcitation.

In order to deepen the insight into the relaxation dynamics from the excited state, Figure 2 summarizes the time-resolved emission measurements carried out on the three nanographenes. For each nanographene, the kinetic trace (λ_exc_ = 350 nm and monitored at the respective emission maxima) is shown at the top of the figure, and panel (a) refers specifically to **c-NG**, (c) to **m-NG** and (e) to **c-Br-NG**. In the case of **c-NG** and **m-NG,** the emission is characterized by a purely single exponential decay, with a lifetime τ of 3.5 and 16.7 ns, respectively. However, this lifetime increase results to be more pronounced than that found for their emission quantum yields, suggesting that the substitution of the oxygen atom with the CH_2_ group produces not only a reduction in the non-radiative decay rate (k_NR_) but also of the radiative one (k_R_). More quantitatively, from the observed QY and lifetimes, we calculated k_R_ = 6 × 10^6^ s^−1^ and k_NR_ = 3 × 10^8^ s^−1^ for **c-NG**, while k_R_ = 3 × 10^6^ s^−1^ and k_NR_ = 6 × 10^7^ s^−1^ for **m-NG**. The small value found for the radiative decay rates confirms that the emission occurs through a weak allowed transition. Moreover, the polar nature of C=O is expected to favor a stronger structural relaxation in the excited state of **c-NG**, hence a higher non-radiative decay probability. The kinetic trace at 460 nm emission of **c-Br-NG** (Figure 2e), unlike the ones of **c-NG** and **m-NG**, reveals that the PL decay for such nanographene is substantially pulse-limited in the early stage, i.e., its time constant is smaller than the temporal resolution given by the instrument response function (dashed black line). 

The lower part of Figure 2 contains information related to the temporal evolution of the emission bandshapes. Indeed, in correspondence with each PL decay trace reported in the top panels, the normalized PL spectra acquired at different delays from photoexcitation are overlayed in panels (b), (d) and (f), referring to **c-NG**, **m-NG** and **c-Br-NG**, respectively. As can be appreciated from panels (b) and (d), no spectral evolution with time was found for **c-NG** and **m-NG**, confirming the occurrence of a synchronous monoexponentially decay of the entire emission band, thus arising from a single electronic state. Differently, Figure 2f highlights the non-trivial relaxation dynamics affecting **c-Br-NG**. Indeed, its emission spectrum in correspondence to the peak of photoexcitation (time delay equals zero) recalls the shape of its steady-state PL, but during a few ns, a complete conversion of the emission to broader, red-shifted and unstructured bands peaking at ~590 nm is registered. The PL decay of this band can be modeled in terms of an exponential decay with a τ=8 ns (see Figure S4). Since we can exclude any inhomogeneity of the sample, as already attested from steady-state characterization, the described dynamics can be explained by an efficient excited-state interconversion between emissive states faster than the temporal duration of the excitation pulse employed in the setup. 

Therefore, our time-resolved emission measurements revealed that moving from **c-NG** to **m-NG,** the substitution of the oxygen with a methylene group has a clear impact on both the radiative and non-radiative decay rate from the first photoexcited state. Instead, moving from **c-NG** to **c-Br-NG,** we found a much more marked variation, determining a deep alteration of the excited state relaxation dynamics, as highlighted by the pulse-limited decay of the PL band and the concurrent transition to another emitting state. By a direct comparison of the structures of **c-NG** and **c-Br-NG**, we can directly ascribe such a behavior to the introduction of the two bromine atoms favoring a heavy atom effect [40,41]. The resulting enhancement of the spin–orbit coupling thus determines an increase in the intersystem crossing rate from singlet state S_1_ to triplet state T_1_. Based on this interpretation, during the early ns-dynamics, the PL undergoes a complete conversion to a phosphorescence emission centered at 2.10 eV. For comparison, theoretical calculations predicted an energy separation of 2.56 eV between S_0_ and T_1_ (details shown in Supplementary Materials, Figures S5 and S6).

**Figure 3:**
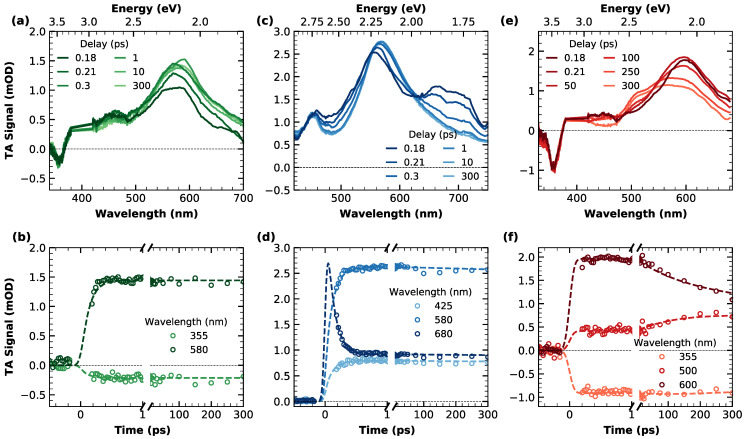
Top panels: transient absorption spectra of (**a**) **c-NG**, (**c**) **m-NG** and (**e**) **c-Br-NG**, acquired at different time delays from photoexcitation ($\lambda_{exc}=400$ nm). Data around 400 nm were removed because of pump scattering. Bottom panels: transient absorption kinetic traces and relative fitting curves cut at different probe wavelengths of (**b**) **c-NG**, (**d**) **m-NG** and (**f**) **c-Br-NG**.

In order to have a complete picture of the faster relaxation processes occurring on the femtosecond and picosecond timescale, the three compounds were investigated by ultrafast pump–probe transient absorption (TA) measurements, photoexciting by ~90 fs pulses at 400 nm. TA data of the three nanographenes are reported in Figure 3. The top panels concern the broadband spectral evolution, while the corresponding kinetic traces are shown on the bottom panels.

In Figure 3a, TA spectra of **c-NG** acquired at different delays from photoexcitation are overlayed. As can be seen, they are characterized by an extended excited state absorption (ESA) dominating the entire spectral range investigated, with a well-defined band peaking at ~580 nm. A clear negative contribution superimposed to the ESA in the region around 360 nm is ascribable to a ground state bleaching (GSB) associated with the depopulation of the ground state by pump absorption, in agreement with the steady-state absorption position. Regarding the temporal evolution, the only dynamics observed are represented by a very fast rise of the ESA, fully completed within the first 200 fs after excitation. The absence of any additional dynamics in the ps-scale can also be inferred from the kinetic traces shown in panel (b) of the same figure. In order to obtain a quantitative analysis of the spectral evolution and the relative time scales, we decomposed the experimental TA data via singular value decomposition and performed a global wavelength-time multiexponential fit: the entire spectral evolution can be described by two decay-associated spectra (DAS) with respective time constants of $\tau_{1}=120\pm 15$ fs and $\tau_{2}>1$ ns (Figure S7). DAS_1_ corresponds to the fast ESA rise, probably compatible with an internal conversion process. Such fast relaxations are very common for photoexcited aromatic molecules, usually characterized by an ultrafast population of the lowest energy singlet excited state S1 from higher energy photoexcited states [42,43]. Indeed, observation of the steady-state absorption in Figure 1 makes it clear that photons at 400 nm (3.1 eV) initially populate an upper excited state, from which an internal conversion is required to populate the emissive state, whose electronic origin can be located around 450 nm (2.75 eV) from the fluorescence spectra. DAS_2_ is instead related to the decay of the overall signal occurring on the ns-scale, consistent with the previously found fluorescence lifetime. 

Figure 3c illustrates the temporal evolution of the broadband TA signal of **m-NG**. Analogously to compound **c-NG**, the positiveness of the TA signal in the 400–750 nm range is due to the presence of a broad ESA, with a main band whose peak evolves in the 550–570 nm range and an additional structured band around 680 nm. The temporal evolution of the signal reveals an isosbestic point between the two ESA bands at ~630 nm, accounting for the decrease in the signal around 680 nm and the concomitant rise and redshift of the main ESA peak to 570 nm. Similarly to what was found for **c-NG**, after such initial dynamics, which is already completed in less than 200 fs, no further evolution of the signal was found in the ps-range, as can be better evinced also from panel (d). Moreover, in this case, a global fitting procedure permitted fully reconstructing the TA data of **m-NG** in terms of two DAS, where the corresponding time constants of $\tau_{1}=120\pm 8$ fs and $\tau_{2}>1$ ns (Figure S8) resulted in being identical to those found for **c-NG**. The shape of DAS_1_ confirms that the initial dynamics are consistent with an internal conversion process, as already found for **c-NG**. As in the previous case, $\tau_{2}$ corresponds to the ns depopulation of the lowest excited state. 

Finally, the right part of Figure 3 is dedicated to the TA data of nanographene **c-Br-NG**. Its spectral shape (Figure 3e) recalls the one found for **c-NG** (Figure 3a), with a GSB negative peak at 360 nm and a broad ESA band initially peaking at 600 nm, but this time the observed dynamics are clearly different. More specifically, a spectral change in the signal is recognizable around an isosbestic point at 550 nm due to the simultaneous decrease in the band at 600 nm and the rise of an ESA contribution around 520 nm. Since this evolution occurs on a scale of hundreds of ps, we can exclude that it is ascribable to internal conversion as in **c-NG** and **m-NG**, but also to any vibrational relaxation or solvation process, which typically lasts up to less than 1 ps [44,45]. Moreover, as also highlighted from the kinetic trace cut at 355 nm (Figure 3f), no GSB dynamics were found, thus ruling out the occurrence of a ground state recovery within this temporal window. By gathering these considerations, we can instead ascribe the ESA component rising at 520 to a triplet-triplet absorption T_1_→T_n_ contribution due to the simultaneous population of T_1_ from S_1_ via an intersystem crossing mechanism. Thus, this interpretation is in perfect agreement with the aforementioned conversion of the emission to a phosphorescence, inferred on the basis of the measurements reported in Figure 2e. TA data of **c-Br-NG** were spectrally decomposed into two DAS with lifetimes $\tau_{1}=150\pm 20$ ps and $\tau_{2}\;≳1$ ns (Figure S9). Again, $\tau_{2}\;$refers to the ns-ground state recovery. Instead, we associated DAS_1_ with the rise of a T_1_→T_n_ transition, indicating the early stage of S_1_→T_1_ intersystem crossing. A time constant of 150 ps for intersystem crossing is clearly consistent with nanosecond-resolved data in Figure 2, which suggests it occurred within the IRF of the nanosecond experiment. 

**Figure 4:**
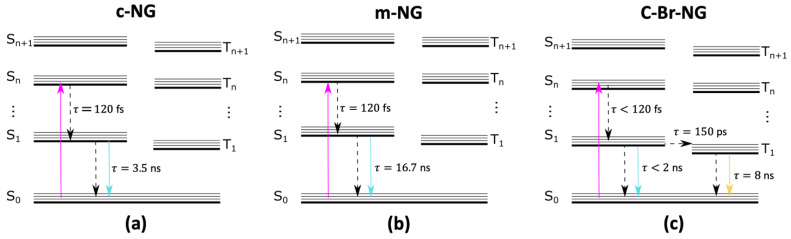
Illustration of the excited-state electronic deactivation pathways and associated timescales of (**a**) **c-NG**, (**b**) **m-NG** and (**c**) **c-Br-NG**.

From the thorough characterization described in Figure 4, we drafted a simplified Jablonski diagram to summarize schematically the relaxation cascades occurring after the photoexcitation of the three compounds. As described, **c-NG** and **m-NG** share substantially the same Kasha-obeying deactivation pathways, involving an internal conversion to the first singlet excited state S_1_, lasting for about 120 femtoseconds, followed by a ground state recovery occurring in 3.5 and 16.7 ns for **c-NG** and **m-NG,** respectively. The two different ns-timescales account for the different impacts that the oxygen and methylene groups have on both the radiative and non-radiative decay rates. If compared to **c-NG**, the addition of two peripherical bromine atoms in **c-Br-NG** causes more significant changes in the relaxation dynamics, mainly characterized by an intersystem crossing on the hundred ps scale, which rapidly depopulates S_1_ and determining a dramatic decrease in the singlet emission quantum yield. 

## 4. Conclusions

In summary, the aim of this work was to investigate the photophysical properties of three atomically precise distorted nanographenes, specifically the three heptagon-containing HBC derivatives **c-NG, m-NG** and **c-Br-NG**, differing from each other for specific modifications of their edge functional groups. In particular, a side-by-side comparative analysis involving spectroscopic steady-state and time-resolved methods permitted us to shed light on the impact that the different edge chemistry of the studied nanographenes exert on their optical photocycle down to the femtosecond scale by establishing well-defined structure–properties correlations. The different edge chemical functionalization has a strong impact on the vibronic coupling of the emissive transition, the photoinduced relaxation dynamics, the radiative and non-radiative rate decay and, clearly, allows for tuning the emission efficiency in a wide range. From a more general point of view, our results asseverate the huge need for designing and studying nanographenes of precise structure to fully disclose the impact of each structural element on the physico-chemical features and reliably control them for future applications. 

## Data Availability

The data presented in this work are available on request from the corresponding author.

## Supplementary Materials

The following supporting information can be downloaded at: https://www.mdpi.com/article/10.3390/ma16020835/s1, Figure S1: Absorption and emission spectra; Figure S2: Excitation-emission contour plot; Figure S3: Absorption/Excitation spectra; Figure S4: Emission decay fit of c-BR-NG; Figure S5: DFT optimized geometry of c-Br-NG in S_0_ state; Figure S6: DFT optimized geometry of c-Br-NG in T_1_ state; Figure S7: Decay Associated Spectra of c-NG; Figure S8: Decay Associated Spectra of m-NG; Figure S9: Decay Associated Spectra of c-Br-NG. Reference [46] are cited in the supplementary materials.
