# Supplementary material for: Atomically Precise Distorted Nanographenes: The Effect of Different Edge Functionalization on the Photophysical Properties down to the Femtosecond Scale
Source: Materials (Basel). 2023 Jan 15;16(2):835. doi: 10.3390/ma16020835 (PMC9867459; doi:10.3390/ma16020835)
Supplement: Supplementary file 1 [file materials-16-00835-s001.zip › materials-2142549-supplementary.pdf]

SUPPORTING INFORMATION

Atomically Precise Distorted Nanographenes: The Effect of Different Edge Functionalization on the Photophysical Properties down to the Femtosecond Scale

Marco Reale [1], Alice Sciortino [1,2], Marco Cannas [1], Ermelinda Maçoas [3], Arthur H. G. David [4], Carlos M. Cruz [4], Araceli G. Campaña [4] and Fabrizio Messina [1,2,*]

[1] Dipartimento di Fisica e Chimica—Emilio Segrè, Università degli Studi di Palermo, Via Archirafi 36, 90123 Palermo, Italy

[2] Advanced Technologies Network Center, Università degli Studi di Palermo, Viale delle Scienze Ed. 18/A, 90128 Palermo, Italy

[3] Centro de Química Estrutural e Institute of Molecular Sciences, Instituto Superior Técnico, Universidade de Lisboa (Portugal), Av. Rovisco Pais 1, 1049-001 Lisboa, Portugal

[4] Department of Organic Chemistry, Unidad de Excelencia de Química (UEQ), Faculty of Sciences, University of Granada, Avda. Fuente Nueva s/n, 18071 Granada, Spain

* Correspondence: fabrizio.messina@unipa.it

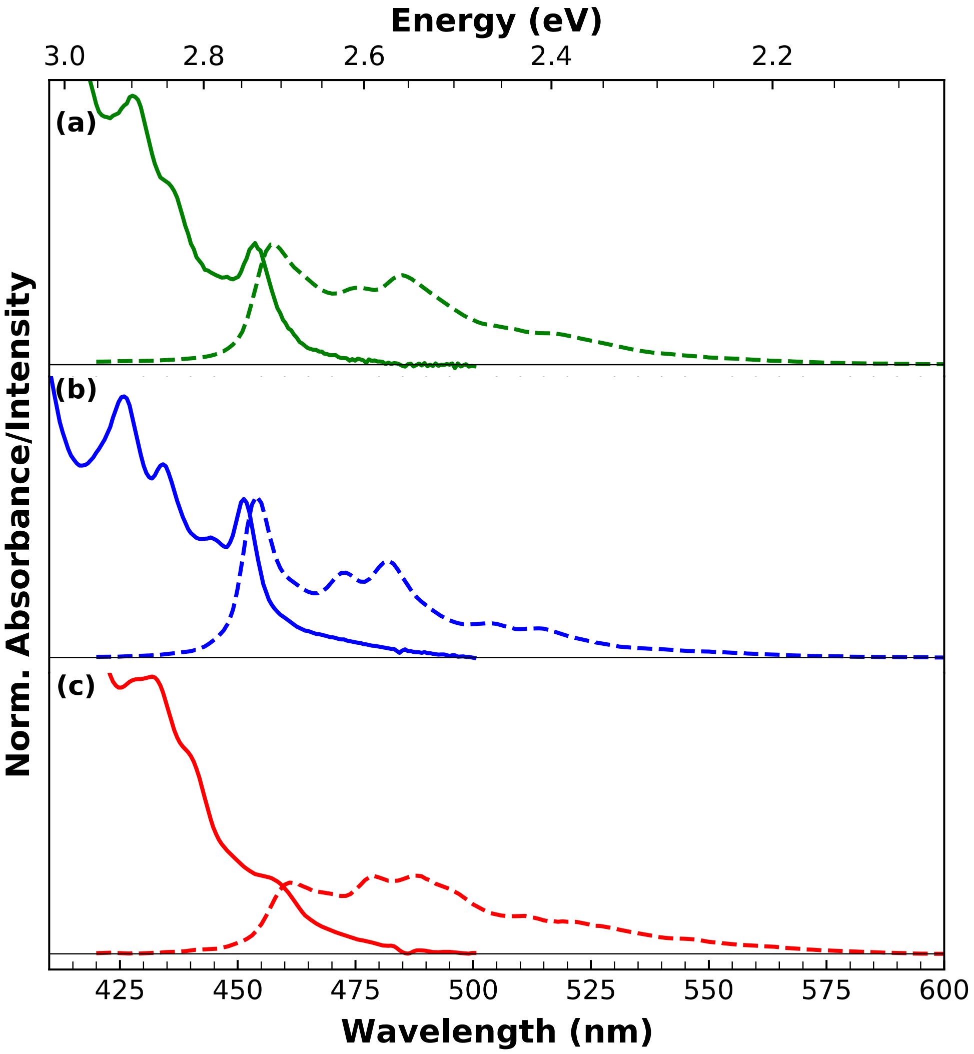

Figure S1 – Comparison of absorption bands (continuous lines) and fluorescence spectra excited at 350 nm (dashed lines) for a) **c-NG**, b) **m-NG** and c) **c-Br-NG**.

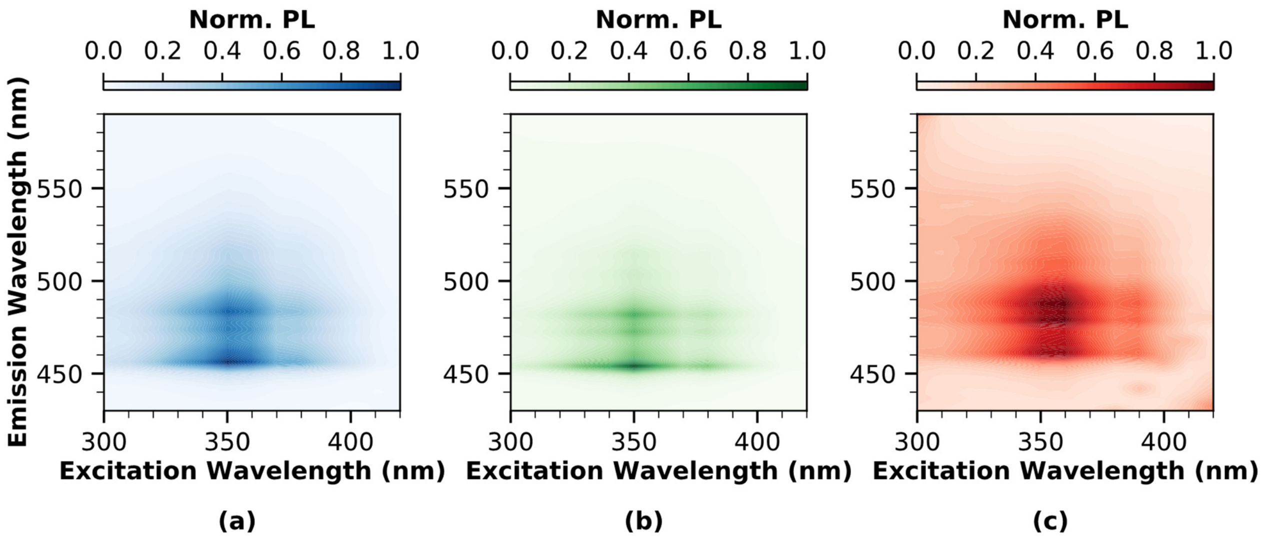

Figure S2 – Excitation-emission map of a**) c-NG**, b) **m-NG** and c) **c-Br-NG**.

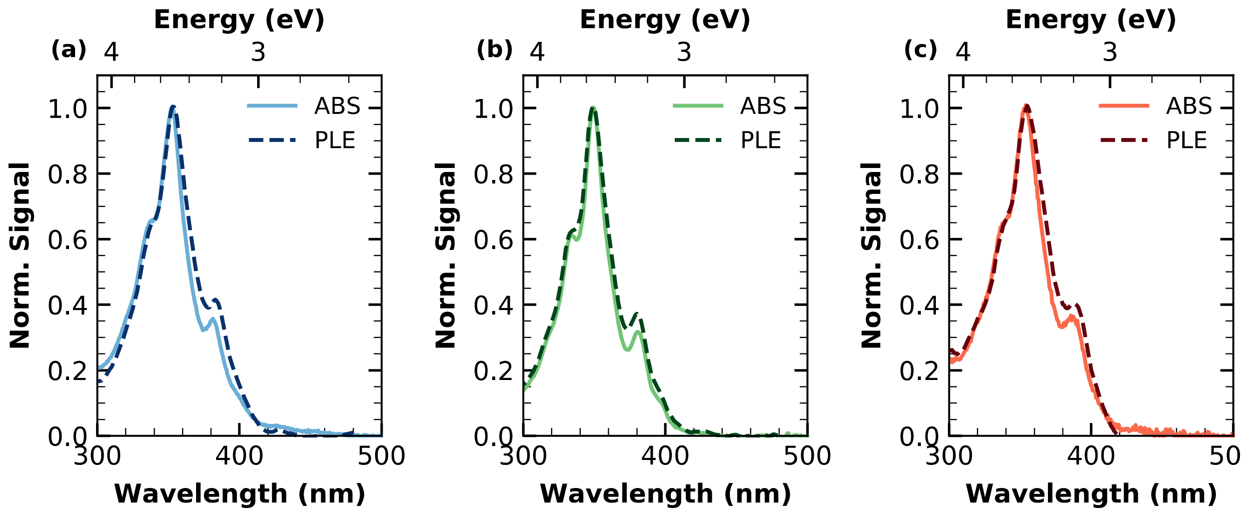

Figure S3 – Comparison of absorption spectrum (ABS, continuous curve) and photoluminescence excitation spectra collected at 490 nm (PLE, dashed curve) of a**) c-NG**, b) **m-NG** and c) **c-Br-NG**

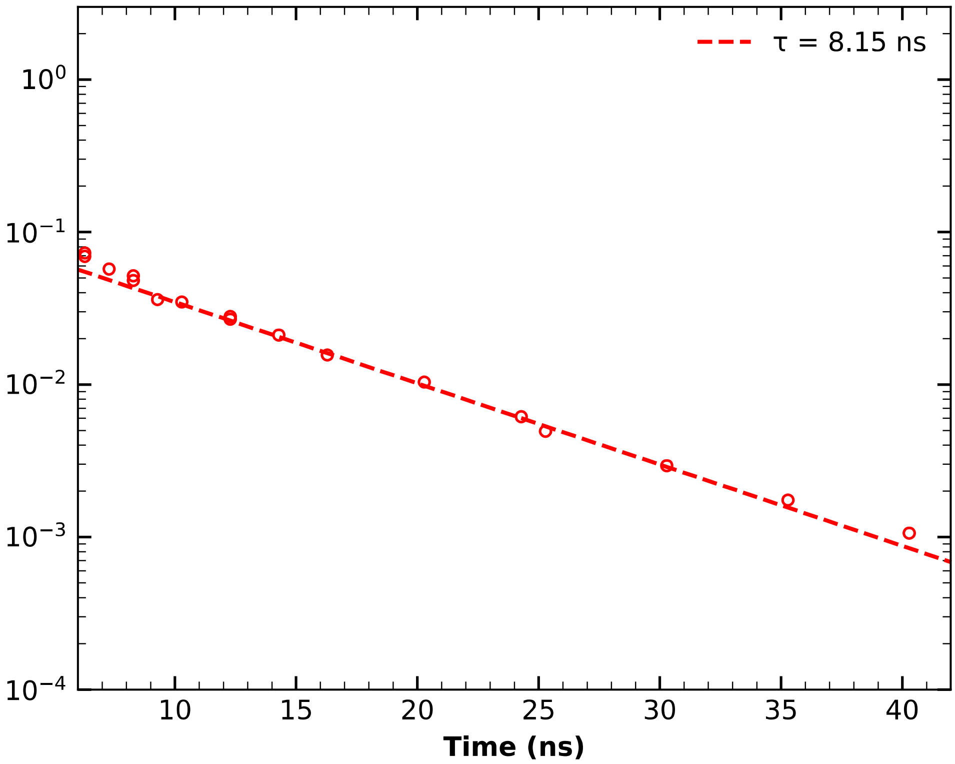

Figure S4 – Exponential fit on the emission decay collected at 590 nm of **c-Br-NG**.

Computational details

Calculations to estimate the energy difference between the singlet ground state and the first triplet excited state of cBrNG were carried out using the Gaussian 16 (revision A.03) software package.[S1] In this case, the two structures were optimized using the B3LYP/6-31G(d,p) level of theory and applying the D3 version of Grimme's dispersion with Becke-Johnson damping (GD3BJ). Vibrational frequencies analysis was performed to ensure that the optimized geometries are stationary points.

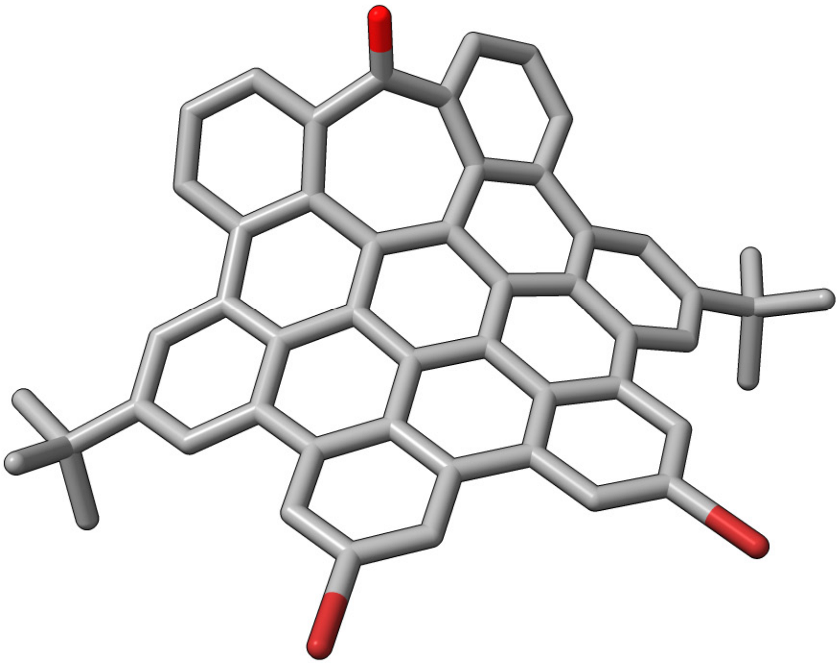

Figure S5. DFT optimized geometry of cBrNG in its S_0 state.

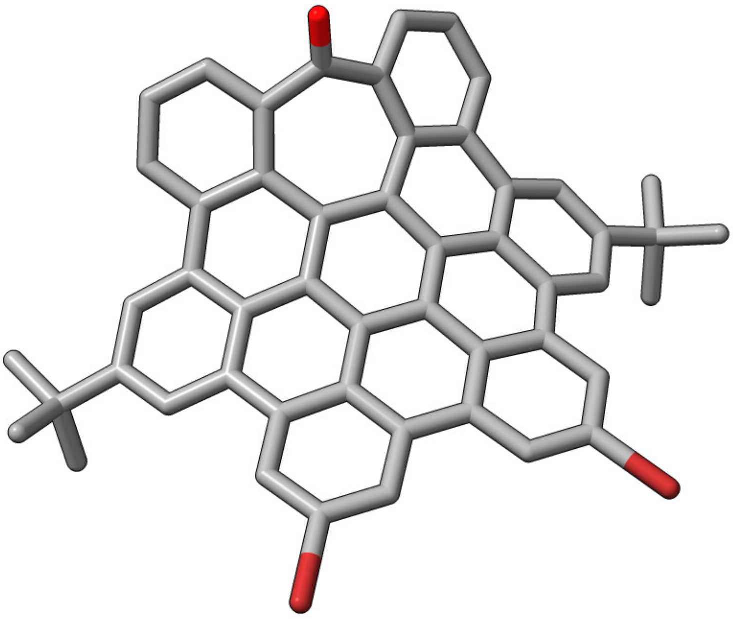

Figure S6. DFT optimized geometry of cBrNG in its T_1 state.

Geometries

c-Br-NG (S_0)

O	-5.14200000	-3.35798100	0.00000000
C	-4.69671100	-2.21863800	0.00000000
C	-4.55260800	-1.45153900	1.25607800
C	-5.64893600	-1.58233200	2.10946600
H	-6.46285500	-2.22768000	1.80111100
C	-5.68510100	-0.90116300	3.31895300
H	-6.55008800	-0.96959600	3.97034000
C	-4.56371600	-0.19438100	3.71151900
H	-4.55106500	0.24724000	4.69910200
C	-3.41356800	-0.11260400	2.90527900
C	-3.42216200	-0.66694800	1.58777200
C	-2.23677300	-0.51152300	0.72127300
C	-1.00516100	-0.26292600	1.40180300
C	-0.97308500	0.10698100	2.79895200
C	-2.17357700	0.38497900	3.48679800
C	-2.12384800	1.00661200	4.74402900
C	-0.92070400	1.28300300	5.38771300
C	0.25830800	0.88625200	4.74917300
C	0.25762100	0.30414100	3.48078000
C	1.50025600	-0.07940700	2.82229800
C	2.69777500	-0.21961100	3.53099200
H	2.72168300	-0.12409000	4.60790800

C	3.86868200	-0.51264100	2.84636700
C	3.89499200	-0.62757300	1.46566300
H	4.84069400	-0.81389600	0.97703300
C	2.70938300	-0.49370100	0.72976200
C	1.48467400	-0.28066800	1.41887100
C	0.23219000	-0.28414000	0.70273000
C	0.23219000	-0.28414000	-0.70273000
C	1.48467400	-0.28066800	-1.41887100
C	2.70938300	-0.49370100	-0.72976200
C	3.89499200	-0.62757300	-1.46566300
H	4.84069400	-0.81389600	-0.97703300
C	3.86868200	-0.51264100	-2.84636700
C	2.69777500	-0.21961100	-3.53099200
H	2.72168300	-0.12409000	-4.60790800
C	1.50025600	-0.07940700	-2.82229800
C	0.25762100	0.30414100	-3.48078000
C	-0.97308500	0.10698100	-2.79895200
C	-1.00516100	-0.26292600	-1.40180300
C	0.25830800	0.88625200	-4.74917300
C	-0.92070400	1.28300300	-5.38771300
C	-2.12384800	1.00661200	-4.74402900
C	-2.17357700	0.38497900	-3.48679800
C	-3.41356800	-0.11260400	-2.90527900
C	-3.42216200	-0.66694800	-1.58777200
C	-2.23677300	-0.51152300	-0.72127300
C	-4.56371600	-0.19438100	-3.71151900
H	-4.55106500	0.24724000	-4.69910200
C	-5.68510100	-0.90116300	-3.31895300
H	-6.55008800	-0.96959600	-3.97034000
C	-5.64893600	-1.58233200	-2.10946600
H	-6.46285500	-2.22768000	-1.80111100
C	-4.55260800	-1.45153900	-1.25607800
H	1.20736200	1.08089000	-5.23240500
H	-3.04863000	1.28505600	-5.22633400
H	1.20736200	1.08089000	5.23240500
H	-3.04863000	1.28505600	5.22633400
C	-0.84721000	1.98341800	6.75048600
C	-0.84721000	1.98341800	-6.75048600
C	-0.14297400	1.05464200	7.76324000
H	0.87432700	0.80670200	7.44812400
H	-0.07943700	1.54058400	8.74252500
H	-0.69522400	0.11725400	7.88099100
C	-2.23832600	2.33328600	7.30282500
H	-2.77992600	3.01277600	6.63725800
H	-2.85062500	1.43898100	7.45628400
H	-2.13379500	2.83206400	8.27081600
C	-0.04014100	3.29175000	6.60375500
H	0.97833400	3.10265400	6.25366500
H	-0.52028100	3.96652800	5.88844500

H	0.02737200	3.80605700	7.56825200
C	-0.14297400	1.05464200	-7.76324000
H	-0.07943700	1.54058400	-8.74252500
H	0.87432700	0.80670200	-7.44812400
H	-0.69522400	0.11725400	-7.88099100
C	-2.23832600	2.33328600	-7.30282500
H	-2.85062500	1.43898100	-7.45628400
H	-2.77992600	3.01277600	-6.63725800
H	-2.13379500	2.83206400	-8.27081600
C	-0.04014100	3.29175000	-6.60375500
H	-0.52028100	3.96652800	-5.88844500
H	0.97833400	3.10265400	-6.25366500
H	0.02737200	3.80605700	-7.56825200
Br	5.49098100	-0.73534600	3.82863100
Br	5.49098100	-0.73534600	-3.82863100

Zero-point correction= 0.673961 (Hartree/Particle)
Thermal correction to Energy= 0.715340
Thermal correction to Enthalpy= 0.716285
Thermal correction to Gibbs Free Energy= 0.600500
Sum of electronic and zero-point Energies= -7181.152939
Sum of electronic and thermal Energies= -7181.111559
Sum of electronic and thermal Enthalpies= -7181.110615
Sum of electronic and thermal Free Energies= -7181.226399

c-Br-NG (T_1)

O	5.21377700	3.32716300	0.00000000
C	4.72901800	2.20261400	0.00000000
C	4.56468000	1.44287500	1.25664700
C	5.65291500	1.57117800	2.11692500
H	6.47244800	2.20994600	1.81019900
C	5.68156800	0.89427400	3.33244600
H	6.54702200	0.96023900	3.98339600
C	4.55884600	0.19480900	3.72608800
H	4.54395100	-0.24549800	4.71386200
C	3.40535100	0.11732000	2.91977800
C	3.42045600	0.66771400	1.58863800
C	2.25066600	0.50531100	0.72649300
C	1.01247700	0.23515200	1.40978100
C	0.97532200	-0.13096600	2.78425100
C	2.17296500	-0.38732700	3.49432900
C	2.11756400	-0.99744000	4.75855400
C	0.90942700	-1.28183400	5.40007600
C	-0.26143400	-0.88599200	4.75284700
C	-0.26193600	-0.30810400	3.47297800
C	-1.49252900	0.09081900	2.83278000
C	-2.69551900	0.23305100	3.54781000
H	-2.72203100	0.15022600	4.62447300

C	-3.86255900	0.52080400	2.84867800
C	-3.90037000	0.63931900	1.46903700
H	-4.84943900	0.82146800	0.98645100
C	-2.71060100	0.50767100	0.71750600
C	-1.47621100	0.30143800	1.41816400
C	-0.23819600	0.29429400	0.71211800
C	-0.23819600	0.29429400	-0.71211800
C	-1.47621100	0.30143800	-1.41816400
C	-2.71060100	0.50767100	-0.71750600
C	-3.90037000	0.63931900	-1.46903700
H	-4.84943900	0.82146800	-0.98645100
C	-3.86255900	0.52080400	-2.84867800
C	-2.69551900	0.23305100	-3.54781000
H	-2.72203100	0.15022600	-4.62447300
C	-1.49252900	0.09081900	-2.83278000
C	-0.26193600	-0.30810400	-3.47297800
C	0.97532200	-0.13096600	-2.78425100
C	1.01247700	0.23515200	-1.40978100
C	-0.26143400	-0.88599200	-4.75284700
C	0.90942700	-1.28183400	-5.40007600
C	2.11756400	-0.99744000	-4.75855400
C	2.17296500	-0.38732700	-3.49432900
C	3.40535100	0.11732000	-2.91977800
C	3.42045600	0.66771400	-1.58863800
C	2.25066600	0.50531100	-0.72649300
C	4.55884600	0.19480900	-3.72608800
H	4.54395100	-0.24549800	-4.71386200
C	5.68156800	0.89427400	-3.33244600
H	6.54702200	0.96023900	-3.98339600
C	5.65291500	1.57117800	-2.11692500
H	6.47244800	2.20994600	-1.81019900
C	4.56468000	1.44287500	-1.25664700
H	-1.21456200	-1.07275200	-5.23171200
H	3.04081700	-1.26369000	-5.25093500
H	-1.21456200	-1.07275200	5.23171200
H	3.04081700	-1.26369000	5.25093500
C	0.83121900	-1.98021300	6.76338100
C	0.83121900	-1.98021300	-6.76338100
C	0.11864600	-1.05396800	7.77270300
H	-0.89771900	-0.80941300	7.45164400
H	0.05049900	-1.53969800	8.75186400
H	0.66697300	-0.11463400	7.89332900
C	2.22172800	-2.32337800	7.32161800
H	2.76962600	-3.00018700	6.65825300
H	2.82909800	-1.42611600	7.47842000
H	2.11585700	-2.82292000	8.28902500
C	0.03015100	-3.29208800	6.61530000
H	-0.98664200	-3.10684400	6.25800300
H	0.51692400	-3.96681300	5.90440200

H	-0.04245700	-3.80478200	7.58038600
C	0.11864600	-1.05396800	-7.77270300
H	0.05049900	-1.53969800	-8.75186400
H	-0.89771900	-0.80941300	-7.45164400
H	0.66697300	-0.11463400	-7.89332900
C	2.22172800	-2.32337800	-7.32161800
H	2.82909800	-1.42611600	-7.47842000
H	2.76962600	-3.00018700	-6.65825300
H	2.11585700	-2.82292000	-8.28902500
C	0.03015100	-3.29208800	-6.61530000
H	0.51692400	-3.96681300	-5.90440200
H	-0.98664200	-3.10684400	-6.25800300
H	-0.04245700	-3.80478200	-7.58038600
Br	-5.48657900	0.73333400	3.82833800
Br	-5.48657900	0.73333400	-3.82833800

Zero-point correction= 0.670256 (Hartree/Particle)
Thermal correction to Energy= 0.712217
Thermal correction to Enthalpy= 0.713161
Thermal correction to Gibbs Free Energy= 0.594889
Sum of electronic and zero-point Energies= -7181.057109
Sum of electronic and thermal Energies= -7181.015148
Sum of electronic and thermal Enthalpies= -7181.014204
Sum of electronic and thermal Free Energies= -7181.132476

References

[S1] Gaussian 16, Revision A.03, M. J. Frisch, G. W. Trucks, H. B. Schlegel, G. E. Scuseria, M. A. Robb, J. R. Cheeseman, G. Scalmani, V. Barone, G. A. Petersson, H. Nakatsuji, X. Li, M. Caricato, A. V. Marenich, J. Bloino, B. G. Janesko, R. Gomperts, B. Mennucci, H. P. Hratchian, J. V. Ortiz, A. F. Izmaylov, J. L. Sonnenberg, D. Williams-Young, F. Ding, F. Lipparini, F. Egidi, J. Goings, B. Peng, A. Petrone, T. Henderson, D. Ranasinghe, V. G. Zakrzewski, J. Gao, N. Rega, G. Zheng, W. Liang, M. Hada, M. Ehara, K. Toyota, R. Fukuda, J. Hasegawa, M. Ishida, T. Nakajima, Y. Honda, O. Kitao, H. Nakai, T. Vreven, K. Throssell, J. A. Montgomery, Jr., J. E. Peralta, F. Ogliaro, M. J. Bearpark, J. J. Heyd, E. N. Brothers, K. N. Kudin, V. N. Staroverov, T. A. Keith, R. Kobayashi, J. Normand, K. Raghavachari, A. P. Rendell, J. C. Burant, S. S. Iyengar, J. Tomasi, M. Cossi, J. M. Millam, M. Klene, C. Adamo, R. Cammi, J. W. Ochterski, R. L. Martin, K. Morokuma, O. Farkas, J. B. Foresman, and D. J. Fox, Gaussian, Inc., Wallingford CT, 2016.

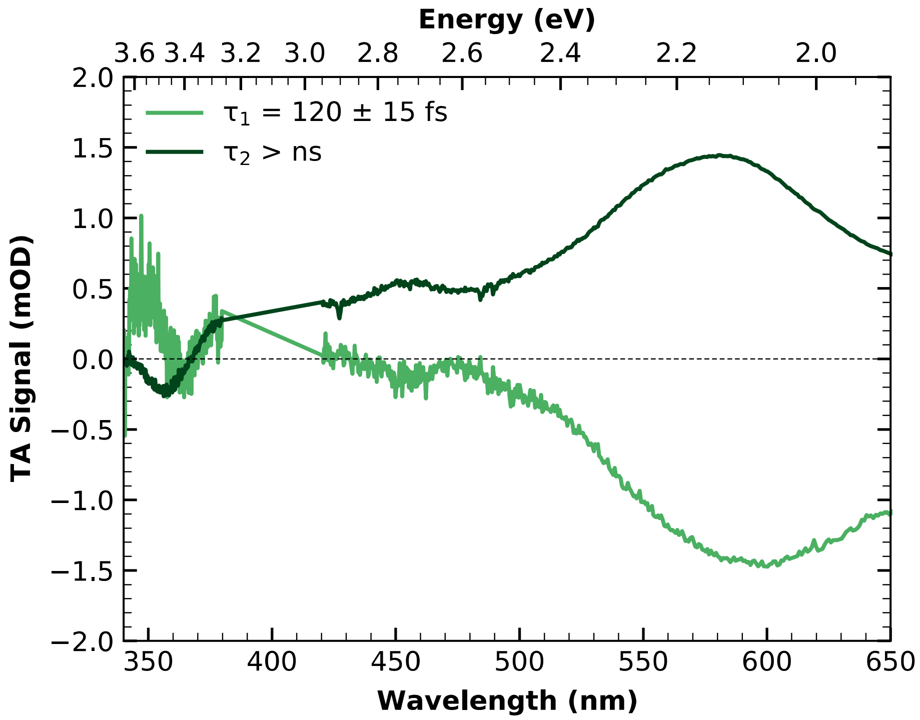

Figure S7 – Decay Associated Spectra decomposition of **c-NG** after SVD analysis with corresponding lifetimes.

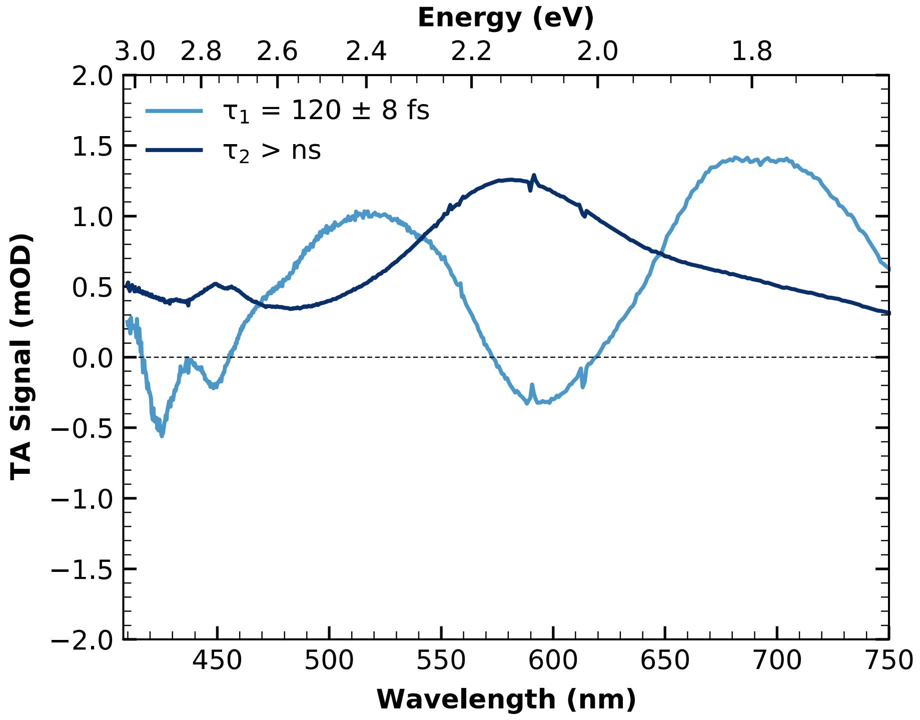

Figure S8 - Decay Associated Spectra decomposition of **m-NG** after SVD analysis with corresponding lifetimes.

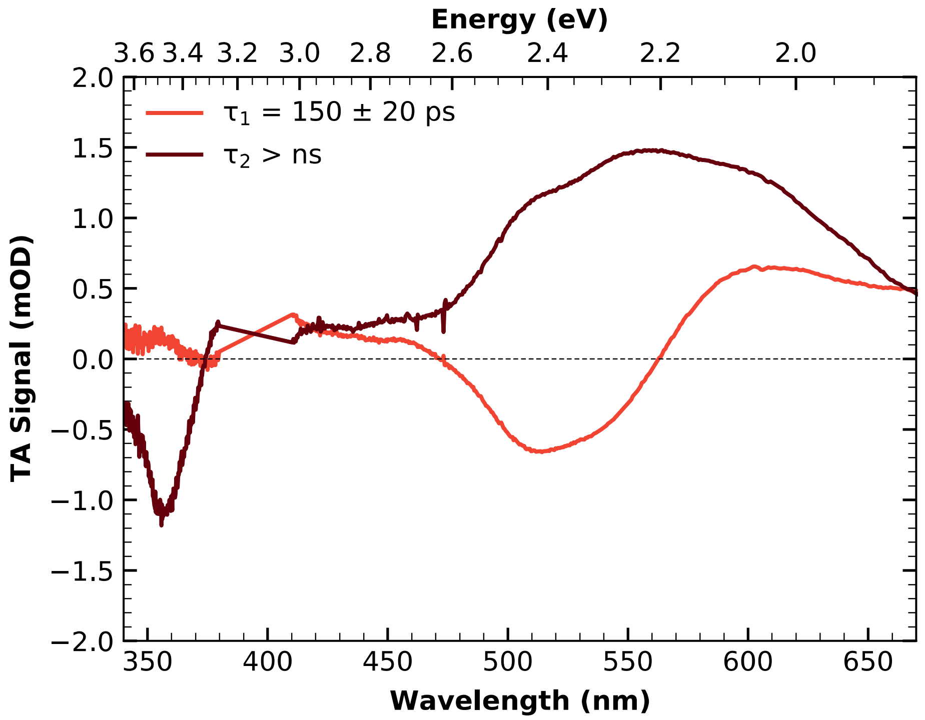

Figure S9 - Decay Associated Spectra decomposition of **c-Br-NG** after SVD analysis with corresponding lifetimes.
